# Molecular epidemiology of pathogenic *Leptospira* spp. in Thailand during 2024–2025, with detection of *Leptospira weilii* in human clinical specimens

**DOI:** 10.1371/journal.pntd.0014720

**Published:** 2026-09-15

**Authors:** Nattapon Nukhonthod, Ifwa Wirasit, Dararat Bangdee, Napaporn Sriden

**Affiliations:** 1 National Institute of Health of Thailand, Department of Medical Sciences, Ministry of Public Health, Nonthaburi, Thailand; 2 Faculty of Medicine, Praboromarajchanok Institute, Ministry of Public Health, Nonthaburi, Thailand; Yale University, UNITED STATES OF AMERICA

## Abstract

**Background:**

Leptospirosis is a significant zoonotic disease in Thailand and globally. The causative agents are the spirochete bacteria in the genus *Leptospira*. An updated assessment of molecular epidemiology in Thailand is needed.

**Methodology/principal findings:**

Remnant EDTA blood specimens from suspected leptospirosis cases collected between June 2024 and July 2025 from the Central, Northern, Northeastern, and Southern regions of Thailand, were referred to the National Institute of Health. Demographic data were collected. Confirmed cases were diagnosed using TaqMan real-time PCR targeting *LipL32* with a positively threshold of Ct < 37. Partial 16S rDNA (approximately 500 bp) was sequenced using Oxford Nanopore (Oxford, UK) and analyze by BLASTn. Among 1,063 suspected cases, 34 (3.2%) were real-time PCR confirmed. The majority of the cases were male (58.8%), primarily residing in Nan province (55.9%), and most positives were from specimens collected during the rainy season (76.5%). Twenty-six of 34 sequences (76.5%) clustered with *L. interrogans*, and 8 of 34 (23.5%) clustered with *L. weilii* within the primary pathogenic (P1) clade. Re-examination with published Thai and regional datasets showed broad circulation of *L. interrogans* and a Northern Thailand–Lao People Democratic Republic (PDR) focus for *L. weilii* To our knowledge, this is the first evidence of *L. weilii* detected in human clinical specimens in Thailand.

**Conclusion/significance:**

Pathogenic *L. interrogans* and *L. weilii* caused human Leptospirosis in Thailand during 2024–2025. These findings update the epidemiological status of *Leptospira* spp. in Thailand and can guide surveillance and control.

## Author summary

Leptospirosis is a zoonotic disease caused by pathogenic *Leptospira* spp., which is endemic in Thailand. Understanding the current molecular epidemiology of *Leptospira* spp. is important in public health intervention and disease control. In this study, we analyzed 1,063 suspected leptospirosis cases collected nationwide during 2024–2025. Thirty-four cases were confirmed by real-time PCR. The majority of cases were from Nan province, which is located in the Northern region of Thailand, and were collected during the rainy season (June – October). Genotypic analysis showed that 26 of 34 cases (76.5%) were infected with *Leptospira interrogans*, while 8 of 34 cases (23.5%) were infected with *Leptospira weilii*. *L. interrogans* and *L. weilii* are classified in primary pathogenic species that cause Leptospirosis. Specifically in Thailand, *L. weilii* has previously been identified in animal clinical specimens and environmental samples, but not in human clinical specimens. To our knowledge, this represents the first report of *L. weilii* detected in human clinical specimens in Thailand.

## Introduction

Leptospirosis is an important zoonotic disease caused by infection with pathogenic *Leptospira spp.*. Multiple mammals serve as potential reservoir hosts, with *Rattus norvegicus* considered as the primary reservoir [1].The estimated global incidence of Leptospirosis is approximately 1.03 million cases per year, with particularly high rates observed in tropical and subtropical countries, especially in Southern and Southeast Asia [2]. In Thailand, the incidence of human Leptospirosis in 2024 was reported 7.18 cases per 100,000 population, which exceeds the median incidence observed over the previous five years [3]. Most cases occur among agricultural workers, who are at increased risk of exposure to contaminated environments, particularly during the rainy season from mid-May to mid-October [3].

Due to nonspecific early symptoms, most leptospirosis cases are often underdiagnosed [4,5]. Leptospirosis incubation period typically ranges from 2 to 30 days after exposure [5,6]. Antibodies usually become detectable approximately 5–14 days after symptom onset, which is the optimal window for indirect diagnosis methods such as immunological and serological assays [7,8]. In addition to the indirect methods, diagnostic tests for leptospirosis can also be direct, including bacterial cultures, microscopy and polymerase chain reaction (PCR) [7,8]. Unless PCR is utilized, isolating *Leptospira sp.* from clinical specimens is tedious, time-consuming and requires technical expertise which is not always practical for immediate treatment decisions [9]. For leptospirosis, both partial and full amplification of 16S rDNA for nucleotide sequencing are the promising approaches for identifying *Leptospira sp.* using advanced molecular techniques. Beyond diagnosis, understanding species biodiversity and distribution is crucial for disease prevention and response [10].

*Leptospira sp.* are spirochetes classified into pathogenic (P) and the saprophytic (S) clades. The pathogenic clade is further divided into two subclades: the primary pathogenic subclade (P1) and the intermediate pathogenic subclade (P2) [11]. Previous studies in Thailand have reported the isolation and detection of various *Leptospira* species from different sources across the country. For instance, the pathogenic species *L. interrogans* (P1) was isolated using culture-based methods from clinical specimens, including kidney samples and patients’ blood [12–14]. Additionally, another P1 species, *L. borgpetersenii*, along with *L. interrogans*, was detected by partial amplification of the 16S rRNA gene from kidney specimens of rodents [15]. For the P2 subclade, *L. wolffii* serovar Korat, was isolated from rodent urine, and its species was confirmed by 16S rDNA sequencing [15]. *Leptospira* spp. was also presented in the environment with previous research successfully isolating both pathogenic and saprophytic *Leptospira* spp. from water and soil samples collected from waterfalls in the Western region of Thailand [16]. The environmental isolates included two P1 species, *L. kmetyi* and *L. tipperaryensis*, as well as the P2 species *L. wolffii* and two saprophytic species, *L. meyeri* and *L. idonii*. Additionally, another study reported the detection of *L. interrogans* and *L. weilii* in environmental samples from the Northern region of Thailand [14].

Taken together, these isolates indicate that *Leptospira* spp. are widely distributed throughout the country; however, there has not been a comprehensive study on their molecular epidemiology, particularly in clinical specimens. Therefore, the objective of this study was to detect and identify *Leptospira* spp. in suspected clinical specimens. Additionally, we investigated the evolutionary relationships of the identified species to enhance our understanding of the epidemiological status of *Leptospira* spp. in Thailand.

## Methods

### Ethic statement

The ethical approval was obtained from the Ethics Committee of Institute for the Development of Human Research Protections (IHRP), Thailand (IHRP No. 25–2568). Data and samples used in this study were accessed on 18/03/2025. The samples collected from the archive were obtained between 2024 and 2025. No new samples were collected from patients; anonymized remnant samples and associated delinked medical histories were used for this molecular epidemiological study.

### Detection of *Leptospira* spp. in clinical specimens by real-time PCR

Total DNA was extracted from EDTA blood specimens using the DNeasy Blood & Tissue Kit following the manufacturer’s instructions (QIAGEN, California, USA). The presence of *Leptospira* DNA was detected using TaqMan polymerase chain reaction (PCR) targeting the *LipL32* gene [17]. The amplification reactions were optimized for the CFX96 Touch Real-Time PCR Detection System (Bio-Rad, California, USA). The amplification was performed using qPCRBIO Probe Mix (PCR Biosystems, London, UK) in a total reaction volume of 20 µl, which contained 0.4 µM of forward primer (LipL32-45F, 5’-AAG CAT TAC CGC TTG TGG TG-3’) and reverse primer (LipL32-286R, 5’-GAA CTC CCA TTT CAG CGA TT-3’), 0.2 µM of probe (LipL32-189P, FAM-5’-AA AGC CAG GAC AAG CGC CG-3’-BHQ1), and 2 µl of template DNA. The amplification conditions consisted of an initial denaturation step at 95°C for 5 minutes, followed by 40 cycles of denaturation step at 95°C for 15 seconds, and annealing and extension step at 58°C for 30 seconds. Genomic DNA extraction from *L. interrogans* serovar Australis and *L. borgpetersenii* serovar Shermani was used as a positive control, and a no template control was used as a negative control to monitor the amplification. Samples that showed a Ct value less than 37 cycles were considered positive [17].

### Amplification of partial 16S rDNA for DNA sequencing

A conventional PCR was conducted to amplify partial 16S rDNA in positive cases. The amplification was done using KOD One PCR Master Mix -Blue- (TOYOBO, Tokyo, Japan) in a total reaction of 20 ul which contained 0.75 uM of forward primer (rrs2_F, 5’-CAT GCA AGT CAA GCG GAG TA-3’) and reverse primer (rrs2_R, 5’-AGT TGA GCC CGC AGT TTT C-3’) [18]. Amplicons were visualized on 2% agarose gel electrophoresis in TBE buffer, followed by staining with SafeView FireRed (Applied Biological Materials Inc., Canada) to confirm the expected size of approximately 541 bp.

### DNA library preparation and sequencing

Library preparation was conducted using the Rapid Barcoding Kit SQK-RBK114.24 (Oxford Nanopore Technologies, Oxford, UK) following the manufacturer’s protocol. In brief, approximately 200 ng of each partial 16S rDNA amplicon was individually barcoded. The barcoded DNA samples were pooled and purified using AMPure XP beads according to the manufacturer’s protocol (Beckman Coulter, California, USA). The purified barcoded DNA was then ligated to the sequencing adapters. Finally, the library was loaded onto a MinION R10.4.1 flow cell (FLO-MIN114) and was sequenced on a Mk1C device (Oxford Nanopore Technologies, Oxford, UK) until an average depth coverage of 100X was achieved.

### Sequencing data analysis

Basecalling and demultiplexing were performed using MinKNOW software v25.05.14, pre-installed in the Mk1C device [19]. Sequencing read quality was assessed using NanoPlot v1.44.1 [20]. Adapter sequences were removed using Dorado v1.0.2, and PCR primer sequences were trimmed with Cutadapt v5.1 [21,22]. Reads with a minimum quality score of Q9 and a maximum read length of 600 bp, which corresponds to the expected amplicon size of approximately 500 bp, were retained. Filtered reads were mapped to the *Leptospira* 16S rRNA reference sequence (NR_180240) using Minimap2 v2.30 with the --ax map-ont option, optimized for Nanopore reads [23]. Mapped reads were indexed and calculated the genome coverage using Samtools v1.22.1, and subsequently generated consensus sequence using Bcftools v1.22 [24,25].

### Phylogenetic analysis

Reference sequences were obtained from NCBI nucleotide database (S1 and S2 Tables). Sequence alignment was performed using Clustal W v2.1 [26] and visualized in MEGA12 (https://www.megasoftware.net). The sequences were trimmed at the 5' and 3' ends to maintain consistent length. A maximum likelihood phylogenetic tree with 1,000 bootstrap replicates was constructed using RAxML-NG v1.2.2 [27], using the best-fit substitution model predicted by ModelTest-NG v0.1.7 [28]. The resulting tree was visualized using the Interactive Tree of Life (iTOL) tool version 7.2.1 [29].

## Results

### Demographic characteristics of laboratory-confirmed leptospirosis cases

A total of 1,063 remnant EDTA blood specimens from suspected leptospirosis cases during 2024–2025 were collected from the Central, Northern, Northeastern, and Southern regions of Thailand and referred to the National Institute of Health of Thailand. Among these specimens, 34 (3.2%, Table 1) were laboratory-confirmed cases of leptospirosis by real-time PCR (referred to as “confirmed cases” for the rest of this study). Most confirmed cases were male (20/34, 58.8%). There was no significant difference in the confirmed cases based on gender (p-value = 1).

**Table 1:** Demographic characteristics of the laboratory-confirmed leptospirosis cases in Thailand during 2024-2025 in this study. Bold numbers indicate statistical significance.

Variable	N (%)	No. of confirmed case (%)	OR (95% CI)	*p*-value
Total	1063	34 (3.2)		
**Gender**				
Male	619 (58.23)	20 (58.8)	1.00 (0.50-2.05)	1
Female	435 (40.92)	14 (41.2)	Reference	
No informed	9 (0.85)	0	–	–
**Age Distribution**				
Children and Teenagers (Under 20)	180 (16.93)	8 (23.53)	1.55 (0.64-3.36)	0.42
Young Adult (21–40)	224 (21.07)	10 (29.41)	1.60 (0.72-3.32)	0.32
Middle Age (41–60)	334 (31.42)	12 (35.29)	1.20 (0.56-2.44)	0.76
Elderly (>60)	320 (30.10)	4 (11.76)	**0.31 (0.09-0.80)**	**0.03**
No informed	5 (0.47)	0	–	–
**Province**				
Nan	545 (51.27)	19 (55.88)	1.21 (0.60-2.45)	0.71
Surat Thani	244 (22.95)	10 (29.41)	1.43 (0.64-2.96)	0.48
Udon Thani	149 (14.07)	2 (5.88)	0.40 (0.06-1.35)	0.25
Chiang Rai	38 (3.57)	1 (2.94)	0.92 (0.03-4.45)	1
Chachoengsao	1 (0.09)	1 (2.94)	–	–
Saraburi	1 (0.09)	1 (2.94)	–	–
Others	85 (7.99)	0	–	–
**Area**				
Rural	693 (65.19)	24 (70.58)	1.28 (0.62-2.85)	0.62
Urban	370 (34.81)	10 (29.41)	Reference	
**Season**				
Hot Season (Mar-May)	280 (26.34)	7 (20.59)	0.73 (0.29-1.62)	0.56
Rainy Season (Jun-Oct)	580 (54.56)	26 (76.47)	**2.75 (1.28-6.60)**	**0.01**
Cold Season (Nov-Feb)	203 (19.10)	1 (2.94)	**0.14 (0.01-0.66)**	**0.03**

The median age of confirmed cases was 34 years (IQR: 21–51 years). Most were of middle-age (41–60 years, 12/34, 35.3%), followed by young adult (21–40 years, 10/34, 29.4%), children and teenagers (under 20 years, 8/34, 23.5%), and elderly (over 60 years, 4/34, 11.8%), respectively (Table 1). The majority of confirmed cases were referred from Nan province (19/34, 55.9%), located in Northern Thailand. This was followed by the cases referred from Surat Thani province (10/34, 29.4%), located in Southern Thailand. Although the confirmed cases were from different regions, most individuals resided in rural areas (24/34, 70.6%). However, there was no significant association between rural area and the occurrence of the confirmed case (p-value = 0.62, Table 1). Confirmed cases primarily occurred from June to October (26/34,76.5%), which is the rainy season in Thailand, according to the Thai Meteorological Department (https://www.tmd.go.th). This was followed by the period from March to June (hot season, 7/34, 20.6%) and from November to February (cold season, 1/34, 2.9%). Univariate logistic regression analysis showed that there was a strong association between rainy season and the occurrence of the confirmed cases (OR: 2.75; 95% CI: 1.28-6.60, Table 1)

### Molecular identification of *Leptospira* spp. in confirmed cases

**Fig 1:**
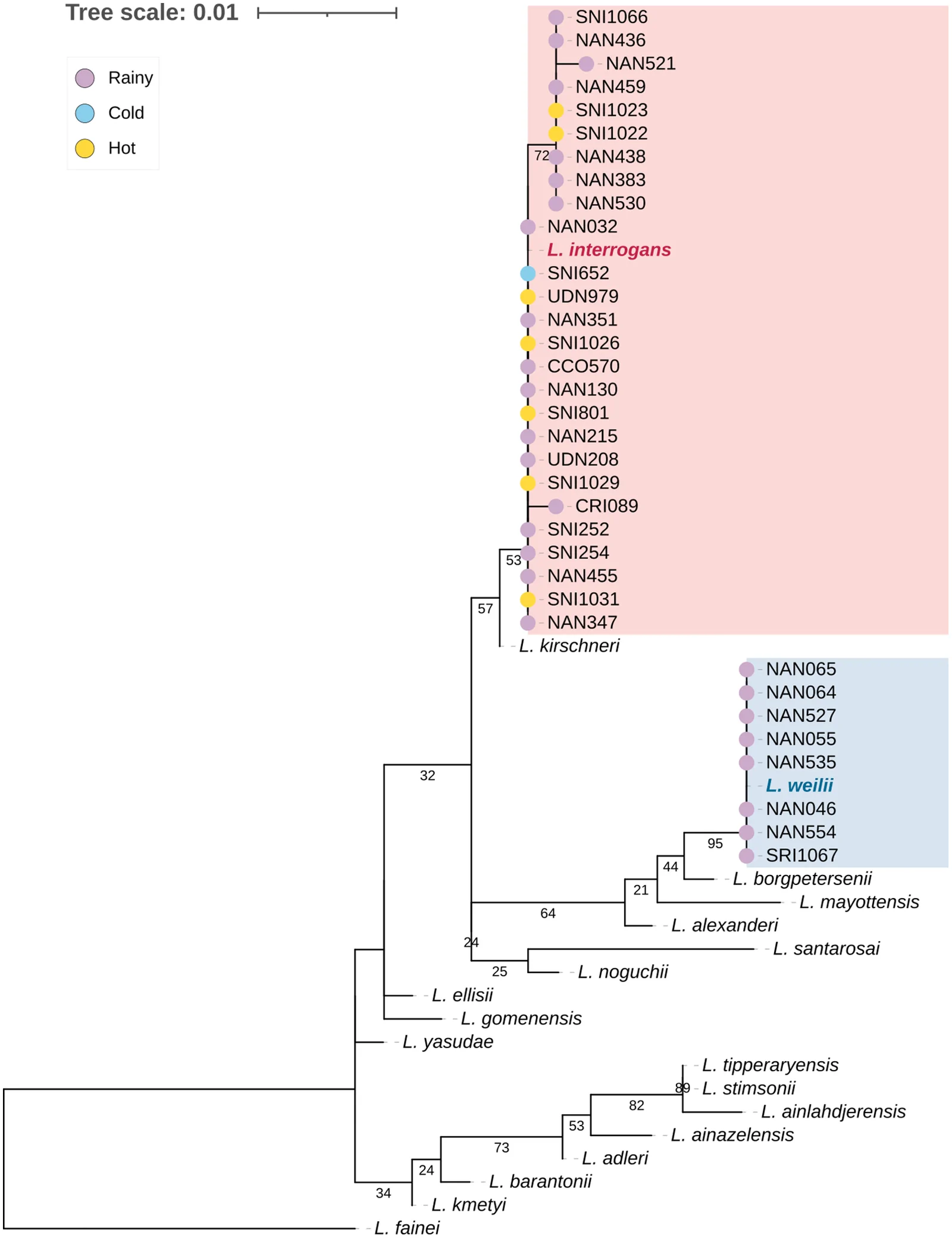
Phylogenetic analysis of partial (499 bp) 16S rDNA sequences acquired in this study together with pathogenic (P1) *Leptospira* spp. obtained from NCBI database. *L. fainei* (P2 species) was used as an outgroup. The three letter code stands for province; CCO (Chachoengsao), CRI (Chiang Rai), NAN (Nan), SNI (Surat Thani), SRI (Saraburi) and UDN (Udon Thani).

Partial 16S rDNA sequences were obtained from all 34 confirmed Leptospirosis cases, as described above. The nucleotide sequences of partial 16S rDNA acquired in this study were submitted to GenBank with the accession numbers PX567771 to PX567804. BLAST analysis was utilized on the consensus sequences to identify the closest *Leptospira* species. The results showed that 26 out of the 34 confirmed cases (76.5%) had high sequence similarity to *L. interrogans*, while the remaining 8 confirmed cases (23.5%) showed high sequence similarity to *L. weilii* (Table 2). To gain insight into the phylogenetic relationships of the identified *Leptospira* spp. in confirmed cases, we constructed a maximum likelihood phylogeny using the consensus sequences from 34 confirmed cases, along with reference 16S rDNA sequences of *Leptospira*, including P1, P2, and S species (S1 Table). *Leptonema illini* was used as an outgroup. The results indicated that all the acquired sequences of the confirmed cases belonged to Clade P1, which is the primary pathogenic species (S1 Fig). Among the 34 sequences, 26 sequences clustered in the same clade with *L. interrogans*, while the other 8 sequences clustered with *L. weilii* (Fig 1), which is in agreement with the BLASTn results (Table 2).

**Table 2:** BLASTn analysis of the partial 16S rDNA sequences of confirmed cases.

Sample ID	Length of the consensus sequence (bp)	BLASTn result			
		Closest *Leptospira sp*. from NCBI (% identity)	GenBank accession no.	Query coverage (%)	e-value
NAN032	505	*L. interrogans* (100%)	CP129087.1	100	0
NAN046	507	*L. weilii* (100%)	CP040840.1	100	0
NAN055	507	*L. weilii* (100%)	CP040840.1	100	0
NAN064	506	*L. weilii* (100%)	CP040840.1	100	0
NAN065	502	*L. weilii* (100%)	CP040840.1	100	0
CRI089	506	*L. interrogans* (100%)	CP136243.1	100	0
NAN130	504	*L. interrogans* (100%)	CP129087.1	100	0
UDN208	507	*L. interrogans* (100%)	CP129087.1	100	0
NAN215	504	*L. interrogans* (100%)	CP129087.1	100	0
SNI252	498	*L. interrogans* (99.80%)	CP129087.1	100	0
SNI254	506	*L. interrogans* (100%)	CP129087.1	100	0
NAN347	506	*L. interrogans* (100%)	CP129087.1	100	0
NAN351	505	*L. interrogans* (100%)	CP129087.1	100	0
NAN383	496	*L. interrogans* (100%)	CP039283.1	100	0
NAN436	504	*L. interrogans* (100%)	CP039283.1	100	0
NAN438	505	*L. interrogans* (100%)	CP039283.1	100	0
NAN455	504	*L. interrogans* (100%)	CP129087.1	100	0
NAN459	505	*L. interrogans* (100%)	CP039283.1	100	0
NAN521	505	*L. interrogans* (99.80%)	CP039283.1	100	0
NAN527	499	*L. weilii* (100%)	CP040840.1	100	0
NAN530	506	*L. interrogans* (100%)	CP039283.1	100	0
NAN535	509	*L. weilii* (100%)	CP040840.1	100	0
NAN554	506	*L. weilii* (100%)	CP040840.1	100	0
CCO570	515	*L. interrogans* (100%)	CP129087.1	100	0
SNI652	515	*L. interrogans* (100%)	CP129087.1	100	0
SNI801	521	*L. interrogans* (100%)	CP129087.1	100	0
UDN979	519	*L. interrogans* (100%)	CP129087.1	100	0
SNI1022	521	*L. interrogans* (100%)	CP039283.1	100	0
SNI1023	505	*L. interrogans* (100%)	CP039283.1	100	0
SNI1026	519	*L. interrogans* (100%)	CP129087.1	100	0
SNI1029	519	*L. interrogans* (100%)	MT745829.1	100	0
SNI1031	519	*L. interrogans* (100%)	CP129087.1	100	0
SNI1066	518	*L. interrogans* (100%)	CP039283.1	100	0
SRI1067	520	*L. weilii* (100%)	CP040840.1	100	0

The phylogeny revealed that the *L. interrogans* clade consisted of sequences of confirmed cases from the Northern region (Chiang Rai and Nan provinces), the Northeastern region (Udon Thani province), the Central region (Chachoengsao province), and the Southern region of Thailand (Surat Thani province) (Fig 1). Notably, confirmed cases in this clade were present in every season (Fig 1). For *L. weilii* clade, it was mainly composed of sequences of the confirmed cases from Nan province, except for one case from Saraburi province, where is located in the Central region. In addition, confirmed cases within *L. weilii* clade were specifically presented during rainy season (Fig 1). These findings suggest that there are differences in the spatiotemporal distribution between *L. interrogans* and *L. weilii* in Thailand.

### Re-examining the phylogeny of the pathogenic *Leptospira* spp. in Thailand and neighboring countries based on 16S rDNA sequences

We further investigated the evolutionary relationship of the identified *Leptospira sp.* by re-examining the phylogeny using the acquired sequences of 34 confirmed cases in this study, along with the publicly available pathogenic *Leptospira* 16S rDNA datasets deposited in NCBI database. The selected datasets included 16S rDNA sequences from various sources, such as clinical specimens from human and animal, and environmental samples in Thailand and neighboring countries (S2 Table).

**Fig 2:**
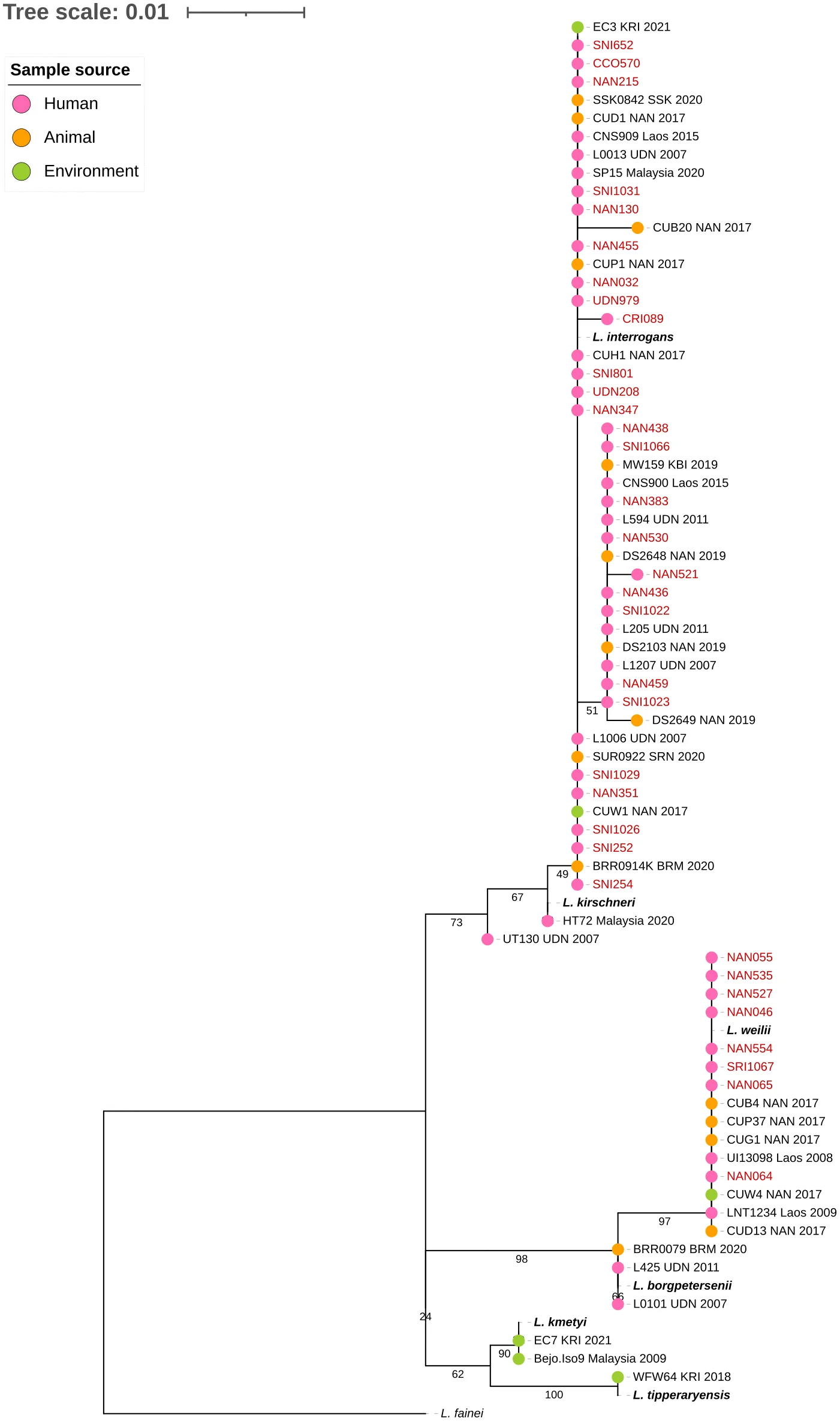
Phylogenetic analysis of partial (403 bp) 16S rDNA sequences acquired in this study together with the previously identified pathogenic (P1) species in Thailand and neighboring countries obtained from NCBI database. *L. fainei* (P2 species) was used as an outgroup. Details of the datasets used to construct the phylogenetic tree are in S2 Table.

**Fig 3:**
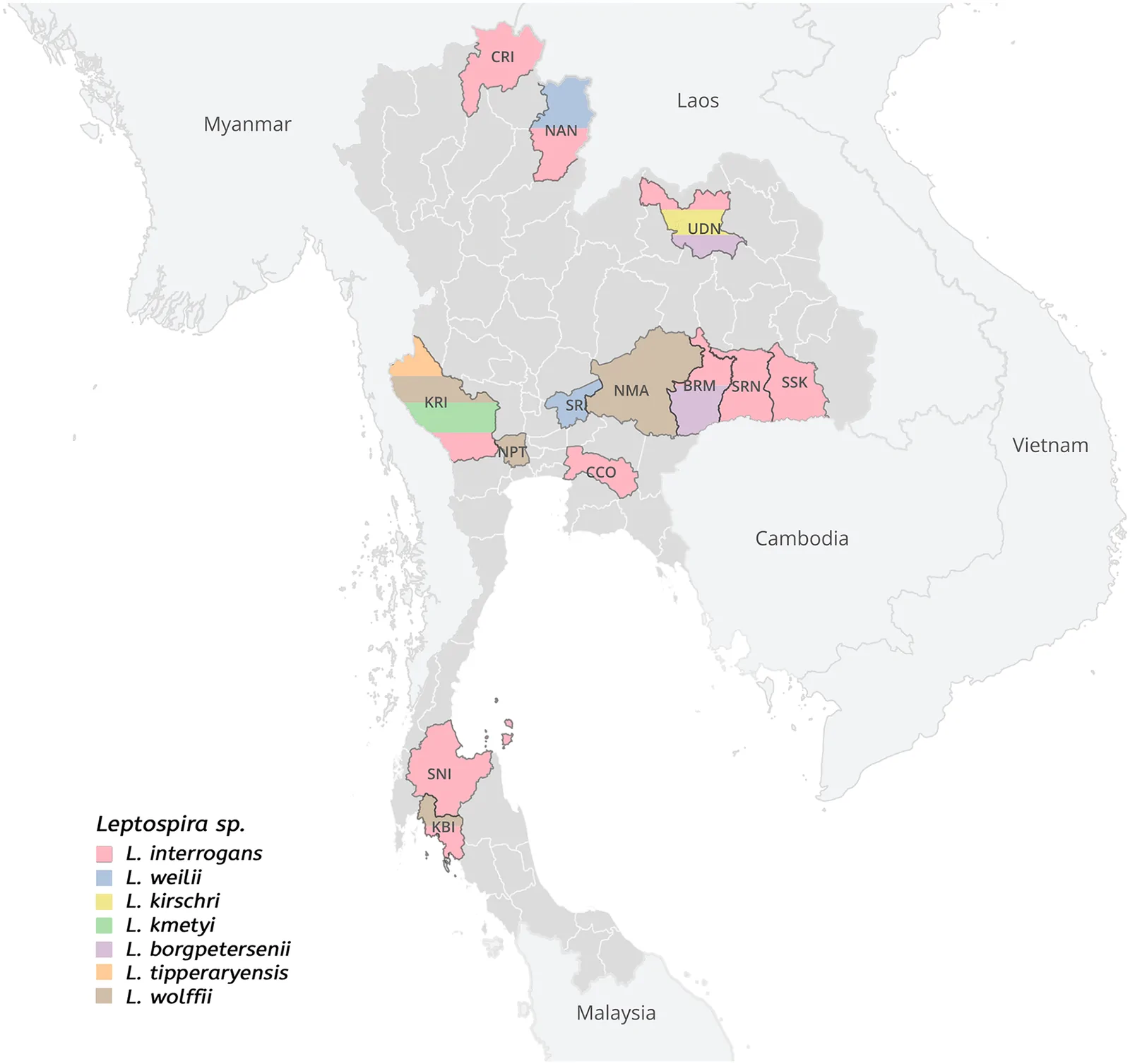
Geographical distribution of pathogenic *Leptospira* spp. identified in the confirmed cases in this study and previously reported in Thailand. Different colors correspond to different *Leptospira sp.* found in each province. The three-letter code stands for province; BRM (Burirum), CCO (Chachoengsao), CRI (Chiang Rai), KBI (Krabi), KRI (Kanchanaburi), NAN (Nan), NMA (Nakhon Ratchasima), NPT (Nakhon Pathom), SNI (Surat Thani), SRI (Saraburi), SRN (Surin), SSK (Sisaket) and UDN (Udon Thani). Map was created in QGIS using shapefiles from geoBoundaries (https://www.geoboundaries.org).

Re-examining the phylogeny revealed that the majority of the pathogenic *Leptospira* in Thailand belonged to *L. interrogans* clade, which contained human, animal and environmental samples from all regions in Thailand, as well as samples from neighboring countries, including Lao PDR and Malaysia (Fig 2). All of 26 sequences in the *L. interrogans* clade acquired in this study (depicted in Fig 1) were also found within this clade with the previously identified *L. interrogans* in country, as well as in Malaysia and Lao PDR (Fig 2). Most of the *L. interrogans* was identified from human samples, while some were identified from animal and environmental samples such as in Nan, Kanchanaburi and Krabi province. To illustrate the landscape of molecular epidemiology, we colored the provinces of origin of the identified *Leptospira sp.* on the map of Thailand (Fig 3). Note that the color in each province does not represent the proportion of a single species, rather it provides the presence of a certain species that had been identified in that province. The *L. interrogans* (colored in red) were identified from various specimens in multiple provinces (Fig 3 and S2 Table). This finding suggested that *L. interrogans* have been circulating throughout the country for over a decade, as the datasets used for the phylogenetic re-examination were deposited between 2007 and 2021.

In the *L. weilii* clade, most sequences originated from the Northern region, specifically Nan Province and Lao PDR (Fig 2). Nan is a border province adjacent to Lao PDR (Fig 3). Additionally, there was no previous study identified *L. weilii* in human clinical specimens in Thailand. Thus, the Thai datasets used for re-examining the phylogeny of *L. weilii* consisted of only animal specimens and environmental samples collected from Nan province (S2 Table). All of the 8 sequences in *L. weilii* clade acquired in this study (depicted in Fig 1) were also found within this clade (Fig 2). Remarkably, this finding reveals that *L. weilii* is also a human pathogenic species in Thailand, marking the first report of this finding.

## Discussion

Leptospirosis is an endemic disease in tropical and subtropical regions worldwide. In Thailand, the first reported case of leptospirosis was documented in 1943 by Yunibandha [30]. Leptospirosis remains one of the important zoonotic diseases that must be reported in Thailand’s Notifiable Disease Surveillance System (R506), in accordance with the Communicable Disease Act of 2015 [3]. The leptospirosis occurs year-round in Thailand, yet it is typically highest during the rainy season or in flooded areas [3,31]. In this study, it was found that the highest incidence of leptospirosis occurs from June to October, aligning with the rainy season as described by the Thai Meteorological Department. Interestingly, previous studies indicated involvement in rice production as the strongest predictor of leptospirosis risk [32,33]. This study also showed that the occurrence of the confirmed cases in men was found higher than in women. Typically, Thai men are more likely to work in agriculture, making them more susceptible to getting infected from the contaminated environment [31].

Understanding of the epidemiological status of pathogenic *Leptospira* spp. plays an essential role in both improving diagnostic methods and epidemiological surveillance of leptospirosis [10]. A recent comprehensive study on the geographical distribution of *Leptospira sp.* illustrated unique patterns in species distribution [10]. Pathogenic species (P1) were widely distributed across the globe. In the Asia-Pacific region, *L. interrogans* were the most prevalent species, followed by *L. borgpetersenii* and *L. kirschneri*, respectively [10]. In Thailand, many pathogenic *Leptospira* spp. were identified and isolated from different sources, with *L. interrogans* being the most predominant species found in human, animal, and environmental samples [12–16,18,34–36]. The landscape of the molecular epidemiology of the identified *Leptospira* spp. in Thailand depicts that *L. interrogans* was the most predominant species found in every region (Fig 3). Interestingly, this study identified *L. weilii* in clinical specimens. Although *L. weilii* ranked lower among the predominant pathogenic *Leptospira sp.* in the Asia-Pacific region with only a few studies having reported *L. weilii* in animal and environmental samples in Thailand [14,37]. Notably, *L. weilii* was also identified from human samples in Lao PDR, which borders the Nan province of Thailand [38]. This study represents the first report of *L. weilii* in human clinical specimens from Nan and Saraburi provinces (Fig 3). Since this study focuses on the molecular epidemiology, further studies on the association between pathogenic *Leptospira* spp. and their clinical manifestations will be crucial for clinicians and researchers to gain a comprehensive understanding of the pathogenicity of Leptospirosis.

## Conclusion

The finding in this study supports and enhances the understanding of the molecular epidemiology of *Leptospira* spp. in Thailand. Additionally, it provides valuable insights into *L. weilii* that have never been previously identified in human clinical specimens in the country. To gain a deeper understanding of the molecular epidemiology, a further study on present of the *Leptospira* spp. in the animals and environment is recommended. More research could also explore the association between these pathogenic species and the severity of the disease using a One Health approach. Taken together, it could contribute to the surveillance and control of the Leptospirosis in Thailand and in the region.

## Supporting information

S1 Fig Phylogenetic analysis of a partial (508 bp) 16S rDNA sequence acquired in this study together with pathogenic (P1 and P2) and saprophytic *Leptospira sp*. obtained from NCBI database. (TIF)

S1 Table Reference *Leptospira* 16S rDNA sequences used in phylogenetic analysis. (DOCX)

S2 Table *Leptospira* 16S rDNA datasets used in phylogenetic re-examination. (DOCX)
