# Supplementary material for: Molecular epidemiology of pathogenic Leptospira spp. in Thailand during 2024–2025, with detection of Leptospira weilii in human clinical specimens
Source: PLoS Negl Trop Dis. 2026 Sep 15;20(9):e0014720. doi: 10.1371/journal.pntd.0014720 (PMC13577409; doi:10.1371/journal.pntd.0014720)
Supplement: Supplementary file 1 [file pntd.0014720.s002.docx]

**S1 Table. Reference *Leptospira* 16S rDNA sequences used in phylogenetic analysis.**

| **Organism** | **GenBank accession no.** | **Identified clade** |
| --- | --- | --- |
| *Leptonema illini* | NR_119299.1 | Outgroup |
| *L. adleri* | MK688976.1 | P1 |
| *L. ainazelensis* | NR_181723.1 | P1 |
| *L. ainlahdjerensis* | NR_181724.1 | P1 |
| *L. alexanderi* | NR_043047.1 | P1 |
| *L. alstonii* | NR_178303.1 | P1 |
| *L. barantonii* | MK791625.1 | P1 |
| *L. borgpetersenii* | NR_043259.1 | P1 |
| *L. ellisii* | MK757997.1 | P1 |
| *L. gomenensis* | MN060987.1 | P1 |
| *L. gorisiae* | NR_199465.1 | P1 |
| *L. interrogans* | NR_116542.1 | P1 |
| *L. kirschneri* | NR_043051.1 | P1 |
| *L. kmetyi* | NR_041544.1 | P1 |
| *L. mayottensis* | NR_134067.1 | P1 |
| *L. noguchii* | NR_043050.1 | P1 |
| *L. santarosai* | NR_115235.1 | P1 |
| *L. stimsonii* | NR_180241.1 | P1 |
| *L. tipperaryensis* | MN062723.1 | P1 |
| *L. weilii* | NR_043044.1 | P1 |
| *L. yasudae* | NR_180240.1 | P1 |
| *L. broomii* | NR_043200.1 | P2 |
| *L. cinconiae* | NR_199464.1 | P2 |
| *L. dzoumogneensis* | MN060997.1 | P2 |
| *L. fainei* | NR_043049.1 | P2 |
| *L. fletcheri* | MN060998.1 | P2 |
| *L. haakeii* | MK791632.1 | P2 |
| *L. hartskeerlii* | MK791631.1 | P2 |
| *L. inadai* | NR_115296.1 | P2 |
| *L. johnsonii* | NR_179210.1 | P2 |
| *L. koniambonensis* | MN061003.1 | P2 |
| *L. licerasiae* | NR_044310.1 | P2 |
| *L. sarikeiensis* | MN062730.1 | P2 |
| *L. semungkisensis* | MN061023.1 | P2 |
| *L. venezuelensis* | MK688379.1 | P2 |
| *L. wolffii* | NR_044042.1 | P2 |
| *L. abararensis* | NR_181721.1 | S |
| *L. bandrabouensis* | MN062727.1 | S |
| *L. biflexa* | NR_043043.1 | S |
| *L. bourretii* | MN061027.1 | S |
| *L. bouyouniensis* | MN061028.1 | S |
| *L. brenneri* | MK791639.1 | S |
| *L. chreensis* | NR_181722.1 | S |
| *L. congkakensis* | MN061030.1 | S |
| *L. ellinghausenii* | NR_179211.1 | S |
| *L. harrisiae* | MK791640.1 | S |
| *L. idonii* | NR_114336.1 | S |
| *L. ilyithenensis* | MN086350.1 | S |
| *L. iowaensis* | NR_199466.1 | S |
| *L. jelokensis* | MN062728.1 | S |
| *L. kanakyensis* | MN061032.1 | S |
| *L. kemamanensis* | MN062726.1 | S |
| *L. kobayashii* | NR_179212.1 | S |
| *L. levettii* | MK791641.1 | S |
| *L. meyeri* | NR_043045.1 | S |
| *L. mgodei* | NR_199467.1 | S |
| *L. milleri* | NR_199468.1 | S |
| *L. mtsangambouensis* | MN062733.1 | S |
| *L. noumeaensis* | MN062729.1 | S |
| *L. ognonensis* | MN082575.1 | S |
| *L. perdikensis* | MN086353.1 | S |
| *L. ryugenii* | NR_179213.1 | S |
| *L. terpstrae* | NR_115294.1 | S |
| *L. vanthielii* | NR_115297.1 | S |
| *L. wolbachii* | NR_043046.1 | S |
| *L. yanagawae* | NR_115293.1 | S |
