# Supplementary material for: Molecular epidemiology of pathogenic Leptospira spp. in Thailand during 2024–2025, with detection of Leptospira weilii in human clinical specimens
Source: PLoS Negl Trop Dis. 2026 Sep 15;20(9):e0014720. doi: 10.1371/journal.pntd.0014720 (PMC13577409; doi:10.1371/journal.pntd.0014720)
Supplement: Supplementary file 2 [file pntd.0014720.s003.docx]

**S2 Table. *Leptospira* 16S rDNA datasets used in phylogenetic re-examination**

| **Name** | **GenBank accession no.** | **Identified clade** | **Source** | **Location** | **Year** | **Reference** |
| --- | --- | --- | --- | --- | --- | --- |
| L1207 UDN 2007 | EF596782.1 | *L. interrogans* | Human | Udon Thani | 2007 | (1) |
| L1006 UDN 2007 | EF536975.1 | *L. interrogans* | Human | Udon Thani | 2007 | (1) |
| L0013 UDN 2007 | EF536978.1 | *L. interrogans* | Human | Udon Thani | 2007 | (1) |
| UT130 UDN 2007 | EF536997.1 | *L. kirschneri* | Human | Udon Thani | 2007 | (1) |
| L0101 UDN 2007 | EF537004.1 | *L. borgpetersenii* | Human | Udon Thani | 2007 | (1) |
| L205 UDN 2011 | JF925165.1 | *L. interrogans* | Human | Udon Thani | 2011 | (2) |
| L594 UDN 2011 | JF925271.1 | *L. interrogans* | Human | Udon Thani | 2011 | (2) |
| L425 UDN 2011 | JF925219.1 | *L. borgpetersenii* | Human | Udon Thani | 2011 | (2) |
| DS2649 NAN 2019 | MK671675.1 | *L. interrogans* | Rodent | Nan | 2019 | (3) |
| DS2648 NAN 2019 | MK671674.1 | *L. interrogans* | Rodent | Nan | 2019 | (3) |
| DS2103 NAN 2019 | MK671673.1 | *L. interrogans* | Rodent | Nan | 2019 | (3) |
| MW159 KBI 2019 | MK370582.1 | *L. interrogans* | Canine | Krabi | 2019 | (4) |
| CNS900 Laos 2015 | KJ150299.1 | *L. interrogans* | Human | Vientiane/ Laos | 2015 | (5) |
| CNS909 Laos 2015 | KJ150300.1 | *L. interrogans* | Human | Vientiane/ Laos | 2015 | (5) |
| CUH1 NAN 2017 | KU854379.1 | *L. interrogans* | Human | Nan | 2017 | (6) |
| CUB20 NAN 2017 | KU854381.1 | *L. interrogans* | Bovine | Nan | 2017 | (6) |
| CUD1 NAN 2017 | KU854375.1 | *L. interrogans* | Canine | Nan | 2017 | (6) |
| CUP1 NAN 2017 | KU854374.1 | *L. interrogans* | Swine | Nan | 2017 | (6) |
| CUW1 NAN 2017 | KU854378.1 | *L. interrogans* | Water | Nan | 2017 | (6) |
| CUB4 NAN 2017 | KU854349.1 | *L. weilii* | Bovine | Nan | 2017 | (6) |
| CUW4 NAN 2017 | KU854372.1 | *L. weilii* | Water | Nan | 2017 | (6) |
| CUP37 NAN 2017 | KU854362.1 | *L. weilii* | Swine | Nan | 2017 | (6) |
| CUG1 NAN 2017 | KU854367.1 | *L. weilii* | Goat | Nan | 2017 | (6) |
| CUD13 NAN 2017 | KU854363.1 | *L. weilii* | Canine | Nan | 2017 | (6) |
| SUR0922 SRN 2020 | KP120912.1 | *L. interrogans* | Rodent | Surin | 2020 | (7) |
| SSK0842 SSK 2020 | KP120907.1 | *L. interrogans* | Rodent | Sisaket | 2020 | (7) |
| BRR0914K BRM 2020 | KP120903.1 | *L. interrogans* | Rodent | Burirum | 2020 | (7) |
| BRR0079 BRM 2020 | KP120895.1 | *L. borgpetersenii* | Rodent | Burirum | 2020 | (7) |
| EC3 KRI 2021 | MT937144.1 | *L. interrogans* | Soil | Kanchanaburi | 2021 | (8) |
| EC7 KRI 2021 | MT937147.1 | *L. kmetyi* | Soil | Kanchanaburi | 2021 | (8) |
| WFW64 KRI 2018 | MF380469.1 | *L. tipperaryensis* | Waterfall | Kanchanaburi | 2018 | (8) |
| SP15 Malaysia 2020 | MK895089.1 | *L. interrogans* | Human | Malaysia | 2020 | (9) |
| HT72 Malaysia 2020 | MK943561.1 | *L. kirschneri* | Human | Malaysia | 2020 | (9) |
| UI13098 Laos 2008 | JQ906673.1 | *L. weilii* | Human | Laos | 2008 | (10) |
| LNT1234 Laos 2009 | JQ906670.1 | *L. weilii* | Human | Laos | 2009 | (10) |
| Bejo/Iso9 Malaysia 2009 | AB279549.1 | *L. kmetyi* | Soil | Malaysia | 2009 | (11) |
| CCO570 | PX567771 | *L. interrogans* | Human | Cha Choeng Sao | 2024 | This study |
| CRI089 | PX567772 | *L. interrogans* | Human | Chiangrai | 2024 | This study |
| NAN032 | PX567773 | *L. interrogans* | Human | Nan | 2024 | This study |
| NAN046 | PX567774 | *L. weilii* | Human | Nan | 2024 | This study |
| NAN055 | PX567775 | *L. weilii* | Human | Nan | 2024 | This study |
| NAN064 | PX567776 | *L. weilii* | Human | Nan | 2024 | This study |
| NAN065 | PX567777 | *L. weilii* | Human | Nan | 2024 | This study |
| NAN130 | PX567778 | *L. interrogans* | Human | Nan | 2024 | This study |
| NAN215 | PX567779 | *L. interrogans* | Human | Nan | 2024 | This study |
| NAN347 | PX567780 | *L. interrogans* | Human | Nan | 2024 | This study |
| NAN351 | PX567781 | *L. interrogans* | Human | Nan | 2024 | This study |
| NAN383 | PX567782 | *L. interrogans* | Human | Nan | 2024 | This study |
| NAN436 | PX567783 | *L. interrogans* | Human | Nan | 2024 | This study |
| NAN438 | PX567784 | *L. interrogans* | Human | Nan | 2024 | This study |
| NAN455 | PX567785 | *L. interrogans* | Human | Nan | 2024 | This study |
| NAN459 | PX567786 | *L. interrogans* | Human | Nan | 2024 | This study |
| NAN521 | PX567787 | *L. interrogans* | Human | Nan | 2024 | This study |
| NAN527 | PX567788 | *L. weilii* | Human | Nan | 2024 | This study |
| NAN530 | PX567789 | *L. interrogans* | Human | Nan | 2024 | This study |
| NAN535 | PX567790 | *L. weilii* | Human | Nan | 2024 | This study |
| NAN554 | PX567791 | *L. weilii* | Human | Nan | 2024 | This study |
| SNI1022 | PX567792 | *L. interrogans* | Human | Surat Thani | 2025 | This study |
| SNI1023 | PX567793 | *L. interrogans* | Human | Surat Thani | 2025 | This study |
| SNI1026 | PX567794 | *L. interrogans* | Human | Surat Thani | 2025 | This study |
| SNI1029 | PX567795 | *L. interrogans* | Human | Surat Thani | 2025 | This study |
| SNI1031 | PX567796 | *L. interrogans* | Human | Surat Thani | 2025 | This study |
| SNI1066 | PX567797 | *L. interrogans* | Human | Surat Thani | 2025 | This study |
| SNI252 | PX567798 | *L. interrogans* | Human | Surat Thani | 2024 | This study |
| SNI254 | PX567799 | *L. interrogans* | Human | Surat Thani | 2024 | This study |
| SNI652 | PX567800 | *L. interrogans* | Human | Surat Thani | 2025 | This study |
| SNI801 | PX567801 | *L. interrogans* | Human | Surat Thani | 2025 | This study |
| SRI1067 | PX567802 | *L. weilii* | Human | Sara Buri | 2025 | This study |
| UDN208 | PX567803 | *L. interrogans* | Human | Udon Thani | 2024 | This study |
| UDN979 | PX567804 | *L. interrogans* | Human | Udon Thani | 2025 | This study |

**References:**

1.	Thaipadungpanit J, Wuthiekanun V, Chierakul W, Smythe LD, Petkanchanapong W, Limpaiboon R, et al. A dominant clone of Leptospira interrogans associated with an outbreak of human leptospirosis in Thailand. PLoS Negl Trop Dis. 2007;1(1):e56.

2.	Boonsilp S, Thaipadungpanit J, Amornchai P, Wuthiekanun V, Chierakul W, Limmathurotsakul D, et al. Molecular detection and speciation of pathogenic Leptospiraspp. in blood from patients with culture-negative leptospirosis. BMC Infectious Diseases. 2011;11(1):338.

3.	Takhampunya R, Korkusol A, Pongpichit C, Yodin K, Rungrojn A, Chanarat N, et al. Metagenomic Approach to Characterizing Disease Epidemiology in a Disease-Endemic Environment in Northern Thailand. Frontiers in Microbiology. 2019;Volume 10 - 2019.

4.	Thongdee M, Chaiwattanarungruengpaisan S, Lekcharoen P, Yimchoho N, Buathong R, Wiriyarat W. A Novel Genotype of Leptospira interrogans Recovered from Leptospirosis Outbreak Samples from Southern Thailand. Jpn J Infect Dis. 2019;72(5):343-6.

5.	Dittrich S, Rattanavong S, Lee SJ, Panyanivong P, Craig SB, Tulsiani SM, et al. Orientia, rickettsia, and leptospira pathogens as causes of CNS infections in Laos: a prospective study. Lancet Glob Health. 2015;3(2):e104-12.

6.	Kurilung A, Chanchaithong P, Lugsomya K, Niyomtham W, Wuthiekanun V, Prapasarakul N. Molecular detection and isolation of pathogenic Leptospira from asymptomatic humans, domestic animals and water sources in Nan province, a rural area of Thailand. Res Vet Sci. 2017;115:146-54.

7.	Krairojananan P, Thaipadungpanit J, Leepitakrat S, Monkanna T, Wanja EW, Schuster AL, et al. Low Prevalence of Leptospira Carriage in Rodents in Leptospirosis-Endemic Northeastern Thailand. Trop Med Infect Dis. 2020;5(4).

8.	Chaiwattanarungruengpaisan S, Suwanpakdee S, Sangkachai N, Chamsai T, Taruyanon K, Thongdee M. Potentially Pathogenic Leptospira Species Isolated from a Waterfall in Thailand. Jpn J Infect Dis. 2018;71(1):65-7.

9.	Philip N, Bahtiar Affendy N, Ramli SNA, Arif M, Raja P, Nagandran E, et al. Leptospira interrogans  and Leptospira kirschneri are the dominant Leptospira species causing human leptospirosis in Central Malaysia. PLoS Negl Trop Dis. 2020;14(3):e0008197.

10.	Boonsilp S, Thaipadungpanit J, Amornchai P, Wuthiekanun V, Bailey MS, Holden MT, et al. A single multilocus sequence typing (MLST) scheme for seven pathogenic Leptospira species. PLoS neglected tropical diseases. 2013;7(1):e1954.

11.	Slack AT, Khairani-Bejo S, Symonds ML, Dohnt MF, Galloway RL, Steigerwalt AG, et al. Leptospira kmetyi sp. nov., isolated from an environmental source in Malaysia. Int J Syst Evol Microbiol. 2009;59(Pt 4):705-8.
